# Evaluation of Antihemagglutinin and Antineuraminidase Antibodies as Correlates of Protection in an Influenza A/H1N1 Virus Healthy Human Challenge Model

**DOI:** 10.1128/mBio.00417-16

**Published:** 2016-04-19

**Authors:** Matthew J. Memoli, Pamela A. Shaw, Alison Han, Lindsay Czajkowski, Susan Reed, Rani Athota, Tyler Bristol, Sarah Fargis, Kyle Risos, John H. Powers, Richard T. Davey, Jeffery K. Taubenberger

**Affiliations:** a Viral Pathogenesis and Evolution Section, Laboratory of Infectious Diseases, Division of Intramural Research, National Institute of Allergy and Infectious Diseases, National Institutes of Health, Bethesda, Maryland, USA; b Perelman School of Medicine, University of Pennsylvania, Philadelphia, Pennsylvania, USA; c Division of Clinical Research, National Institute of Allergy and Infectious Diseases, National Institutes of Health, Bethesda, Maryland, USA; d Clinical Research Section, Laboratory of Immunoregulation, Division of Intramural Research, National Institute of Allergy and Infectious Diseases, National Institutes of Health, Bethesda, Maryland, USA

## Abstract

Despite long-term investment, influenza continues to be a significant worldwide problem. The cornerstone of protection remains vaccination, and approved vaccines seek to elicit a hemagglutination inhibition (HAI) titer of ≥1:40 as the primary correlate of protection. However, recent poor vaccine performance raises questions regarding the protection afforded and whether other correlates of protection should be targeted. A healthy volunteer challenge study was performed with a wild-type 2009 A(H1N1)pdm influenza A challenge virus at the NIH Clinical Center to evaluate two groups of participants with HAI titers of ≥1:40 and <1:40. The primary objective was to determine whether participants with HAI titers of ≥1:40 were less likely to develop mild to moderate influenza disease (MMID) after intranasal inoculation. HAI titers of ≥1:40 were protective against MMID but did not reduce the incidence of symptoms alone. Although the baseline HAI titer correlated with some reduction in disease severity measures, overall, the baseline NAI titer correlated more significantly with all disease severity metrics and had a stronger independent effect on outcome. This study demonstrates the importance of examining other immunological correlates of protection rather than solely HAI titers. This challenge study confirms the importance of NAI titer as a correlate and for the first time establishes that it can be an independent predictor of reduction of all aspects of influenza disease. This suggests that NAI titer may play a more significant role than previously thought and that neuraminidase immunity should be considered when studying susceptibility after vaccination and as a critical target in future influenza vaccine platforms.

## IMPORTANCE

This study represents the first time the current gold standard for evaluating influenza vaccines as set by the U.S. Food and Drug Administration and the European Medicines Agency Committee for Medicinal Products for Human Use, a “protective” hemagglutination inhibition (HAI) titer of ≥1:40, has been evaluated in a well-controlled healthy volunteer challenge study since the cutoff was established. We used our established wild-type influenza A healthy volunteer human challenge model to evaluate how well this antibody titer predicts a reduction in influenza virus-induced disease. We demonstrate that although higher HAI titer is predictive of some protection, there is stronger evidence to suggest that neuraminidase inhibition (NAI) titer is more predictive of protection and reduced disease. This is the first time NAI titer has been clearly identified in a controlled trial of this type to be an independent predictor of a reduction in all aspects of influenza.

## INTRODUCTION

Despite significant investment in influenza preparedness, annual estimates of death due to seasonal influenza range up to 49,000 in the United States (1) and 250,000 to 500,000 deaths in industrialized countries (2, 3). Pandemics can have an even more devastating effect, and continued efforts are being made to improve countermeasures for this worldwide problem (4).

Currently, vaccination is the cornerstone of prophylaxis and the most effective method available to reduce the yearly impact of influenza on the world’s population (5, 6). Influenza vaccines in widespread use are standardized by the amount of the major surface glycoprotein, hemagglutinin (HA), contained in the vaccine preparation. Measurements of serum antibodies in response to the HA are the current gold standard for evaluating vaccines. The U.S. Food and Drug Administration and the European Medicines Agency Committee for Medicinal Products for Human Use both define “protective” titers as a hemagglutination inhibition (HAI) titer of ≥1:40 (7).

The evidence for this cutoff comes from a seminal live influenza virus challenge trial conducted in 1972 by Hobson et al., which established that a prechallenge serum HAI titer of 18 to 36 was associated with 50% protection from infection (8), and a similar study demonstrating a 29% infection rate in those with HAI titers of 1:40 to 60 (9). Other studies in the setting of live and attenuated influenza virus challenge have been less conclusive (10–12). A more recent study, conducted using live attenuated viruses, demonstrated 50% protection from intranasal infection in those with titers of ≥1:40 (13), but a recent epidemiological study of vaccine performance has brought this into question, with only 22% protection of children with postvaccination titers of ≥1:40 (14). Even more concerning are data from the recent influenza seasons suggesting that current seasonal vaccines held to these standards are greatly underperforming, as overall seasonal vaccine effectiveness over the past 10 years has ranged from 10 to 56% with a mean of 40% (15). This is especially concerning for at risk populations who most require protection and in whom even worse performance has been observed (16).

The variability of results in past studies and variations in recent influenza vaccine efficacy are likely multifactorial and suggest that more work is needed to better understand the correlates of protection in influenza infection. Differences in outcome measures, well described interlaboratory variations in HAI and microneutralization assay results (17, 18), evolving viral phenotypes, host factors, and differences in how protection is defined could all further confound interpretation of results and contribute to the variation seen in these and other studies of influenza immunity. Currently, vaccine efficacy is evaluated predominantly by increases in HAI titers, and other possible correlates of immune protection (e.g., neuraminidase inhibition [NAI] titers or other types of immunity) have generally either not been examined or have been relegated to secondary considerations.

This study was designed to evaluate the role of the traditional “protective” HAI titer of ≥1:40 as a correlate of protection in an established well-controlled human challenge study with the influenza A/2009 A(H1N1)pdm (pdm stands for pandemic) virus (19). This is the first healthy volunteer challenge study to specifically evaluate this cutoff since it was established. Virologic and clinical data as correlative parameters of protection were compared between patients with prechallenge titers of ≥1:40 or <1:40. The human challenge model employed in this trial offered a unique opportunity to overcome many of the limitations of past studies and perform not only a comprehensive evaluation of HAI but also to investigate the correlation of antineuraminidase antibodies to protection as measured by baseline neuraminidase inhibition titers. Understanding how these baseline antibody titers predict outcomes as correlates of protection allows us to better understand how different immune factors may interact as independent and dependent factors in influenza disease and may lead to further identification of other correlates of protection, development of better tools to predict outcomes, and identify improved vaccine targets and countermeasures.

## RESULTS

**FIG 1:**
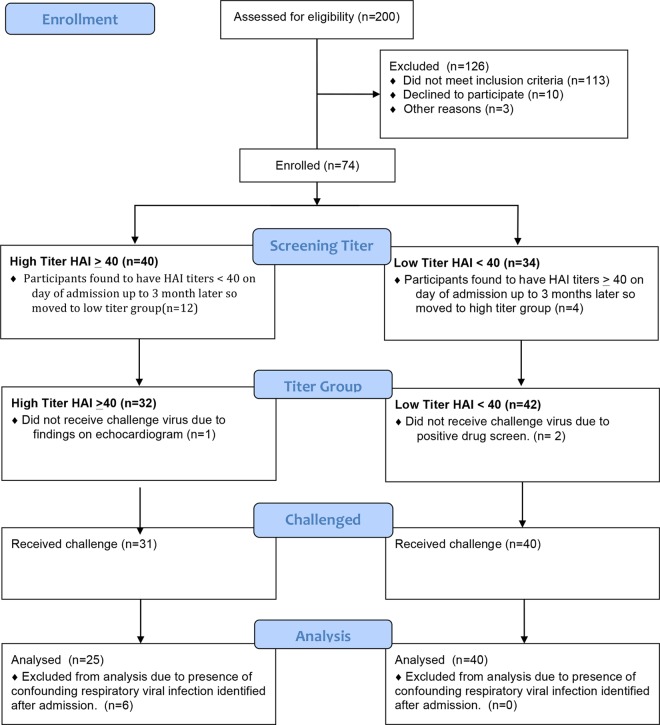
Study flow chart. A total of 200 volunteers were screened for participation. Of these volunteers, 74 were enrolled in the study with 40 in the high-HAI-titer group and 34 in the low-HAI-titer group up to 3 months prior to challenge. On admission for challenge, 16 participants were found to have HAI titers that had increased or decreased since the time of screening either due to waning titers or exposure to virus. Three participants were excluded prior to challenge for safety reasons. Thirty-one participants on the high-titer group and 40 participants in the low-titer group were challenged, and 6 participants in the high-titer group were excluded from the analysis due to detection of another respiratory viral infection within 48 h of challenge.

Seventy-four participants were eligible after screening. As the screening took place up to 3 months in advance, some participants were found to have higher or lower titers at the time of admission so they were moved to the appropriate group for analysis (Fig. 1). After enrollment in the study, three participants were found ineligible for safety reasons and were excluded from challenge, and an additional six were subsequently excluded after testing positive for other respiratory infections within 48 h of influenza virus challenge (Fig. 1). Thus, 65 participants challenged were included in this analysis, 25 participants in the high-titer group with an HAI titer of ≥1:40 (geometric mean titer [GMT] of 214.9) and 40 participants in the low-titer group with an HAI titer of <1:40 (GMT of 6.4). There were no significant differences in age, gender, race, or ethnicity between the two HAI titer groups (Table 1).

**TABLE 1:** Patient baseline characteristics of challenged and eligible subjects by HAI titer group (*n* = 65)

Characteristic	No. of subjects or age (%, unless IQR is specified)	
	High titer (*n* = 25)	Low titer (*n* = 40)
Gender		
Female	14 (56)	19 (48)
Male	11 (44)	21 (52)
Race		
Asian	2 (8)	3 (8)
Black	6 (24)	19 (48)
White	17 (68)	18 (44)
Hispanic or Latino ethnicity		
Yes	4 (16)	3 (8)
No	21 (84)	37 (92)
Age, yr [median (IQR)]^a^	27 (24, 31)	27.5 (24, 30.5)

a IQR, interquartile range.

**FIG 2:**
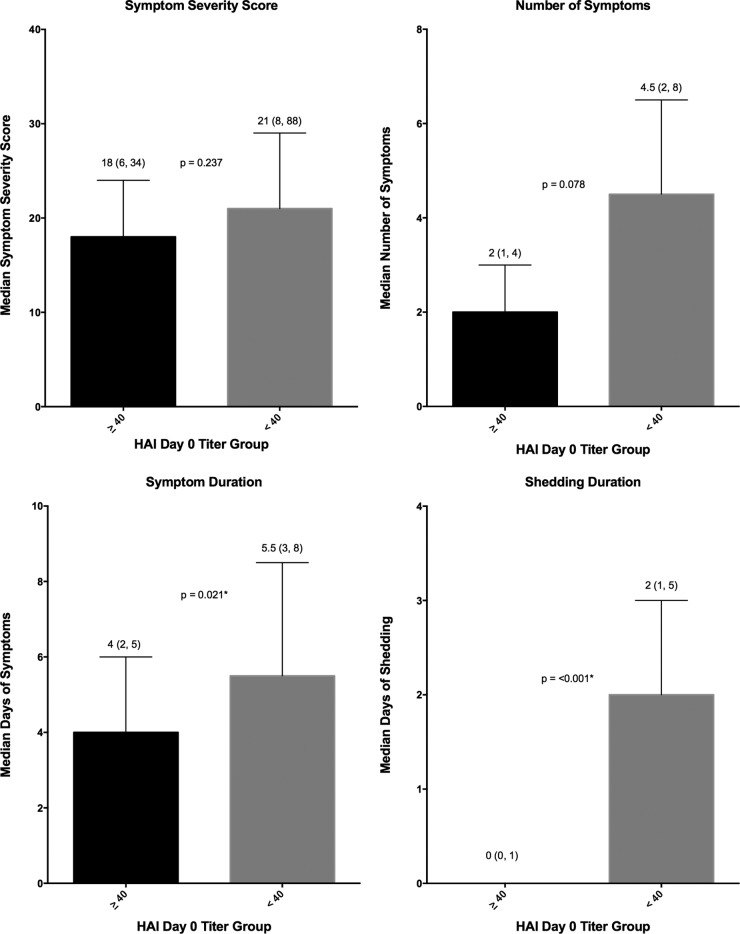
Disease severity measures in high- and low-HAI-titer groups. Symptom severity score (FLU-Pro participant self-assessment), number of symptoms, symptom duration, and shedding duration were evaluated for the participants in the groups with an HAI titer of ≥1:40 or <1:40. The median values (indicated by the bars) are shown as the first value above the bar. The interquartile ranges (indicated by the error bars) are shown in parentheses above the bars. The *P* values generated by the Wilcoxon rank sum test (two sided at 0.05 level) are indicated between the bars for the two HAI titer groups; *P* values that are statistically significant are indicated by an asterisk.

**FIG 3:**
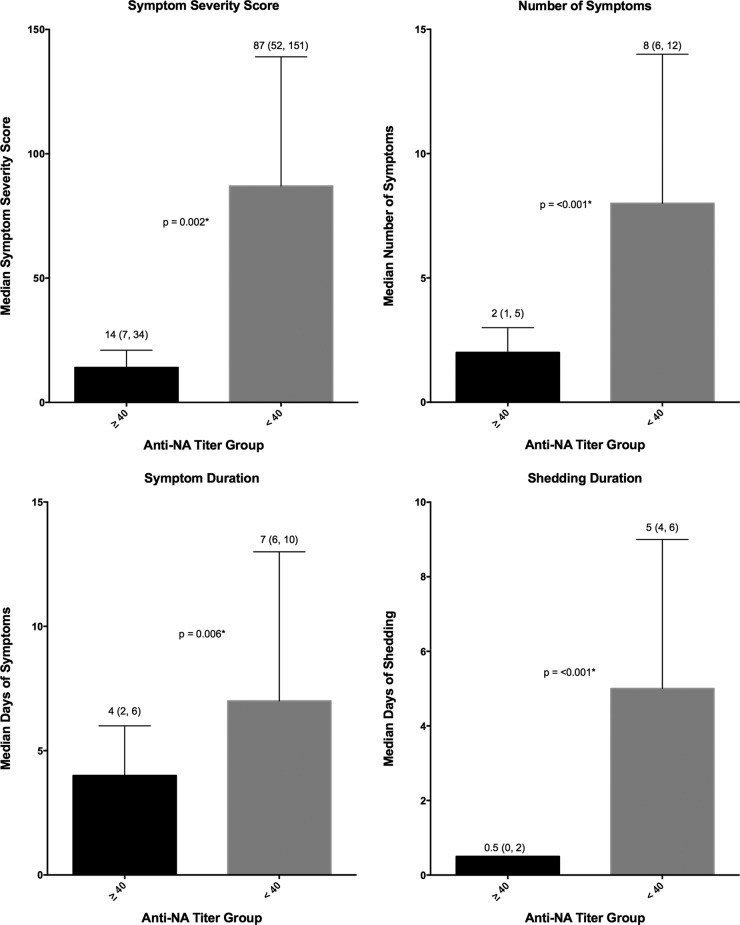
Quantity and timing of viral shedding and composite symptoms. The mean composite symptoms and shedding based on HAI titer groups are shown. Overall, there was primarily a decrease in the duration of shedding and symptoms in those with a higher baseline HAI titer. Values are means ± standard errors of the means (SEM) (error bars).

Significantly fewer participants in the high-HAI-titer group developed MMID than in the low-HAU-titer group (24% versus 72%; *P* < 0.001). However, no statistical difference was observed in the proportion of participants reporting influenza symptoms regardless of viral shedding, with 80% in the high-titer group and 88% in the low-titer group reporting symptoms (*P* = 0.489) (Table 2). Only two of the four disease severity measures, those related to duration of disease (shedding and symptoms) demonstrated a significant decrease in severity in those in the ≥1:40 HAI titer group compared to the <1:40 HAI titer group (Fig. 2). The number and severity of symptoms were not significantly reduced (Fig. 2). Quantitative measurements of viral shedding did demonstrate decreased quantity and duration in the ≥1:40 titer group compared to the <1:40 titer group, which correlated well with reduced symptom duration (Fig. 3).

**TABLE 2:** Primary outcomes by HAI and NAI titer groups

Outcome	No. of subjects (%) with outcome in the HAI titer group		*P* value^a^	No. of subjects (%) with outcome in the NAI titer group		*P* value^a^
	High (*n* = 25)	Low (*n* = 40)		High (*n* = 54)	Low (*n* = 11)	
MMID (shedding + symptoms)	6 (24)	29 (72)	<0.001*	24 (44)	11 (100)	<0.001*
No MMID (shedding + symptoms)	19 (76)	11 (28)		30 (56)	0 (0)	
Symptoms	20 (80)	35 (88)	0.489	44 (81)	11 (100)	0.190
No symptoms	5 (20)	5 (12)		10 (19)	0 (0)	

a Fisher’s exact *P* value for the difference in the number of subjects in the two groups (high- and low-titer groups) (*n* = 65). The statistically significant *P* value is indicated with an asterisk.

**FIG 4:**
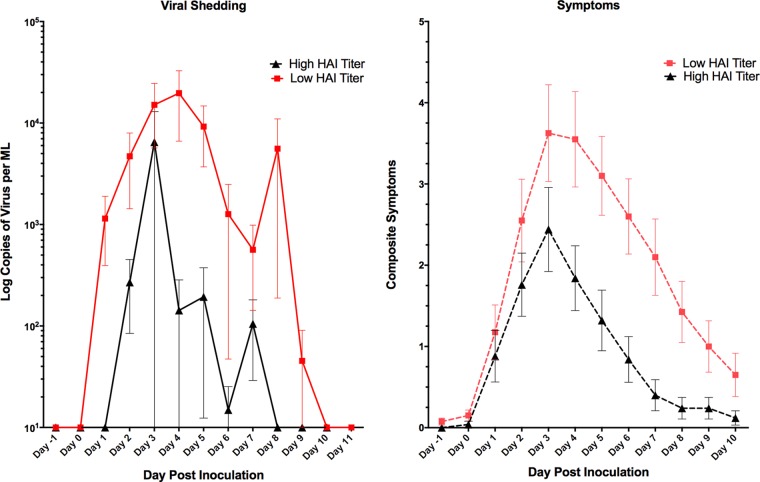
Disease severity measures in high- and low-NAI-titer groups. Symptom severity score (FLU-Pro participant self-assessment), number of symptoms, symptom duration, and shedding duration were evaluated for the participants in the groups with an NAI titer of ≥1:40 and <1:40. The median values (indicated by the bars) are shown as the first value above the bar. The interquartile ranges (indicated by the error bars) are shown in parentheses above the bars. The *P* values generated by the Wilcoxon rank sum test (two sided at 0.05 level) are indicated between the bars for the two NAI titer groups; *P* values that are statistically significant are indicated by an asterisk.

An analysis of these same primary and secondary clinical outcome measures was performed after grouping the participants by baseline (day 0) NAI titer into ≥1:40 (high-titer) or <1:40 (low-titer) groups (Table 1). Similar to baseline HAI, those with a high baseline NAI were found to be less likely to develop MMID, 44% versus 100% (*P* < 0.001), respectively. Unlike baseline HAI titer, all four disease severity metrics demonstrated a reduction in severity of illness in those with NAI titers of ≥1:40, including not only symptom and shedding duration but also reduction in the number of symptoms and symptom severity (Fig. 4).

**FIG 5:**
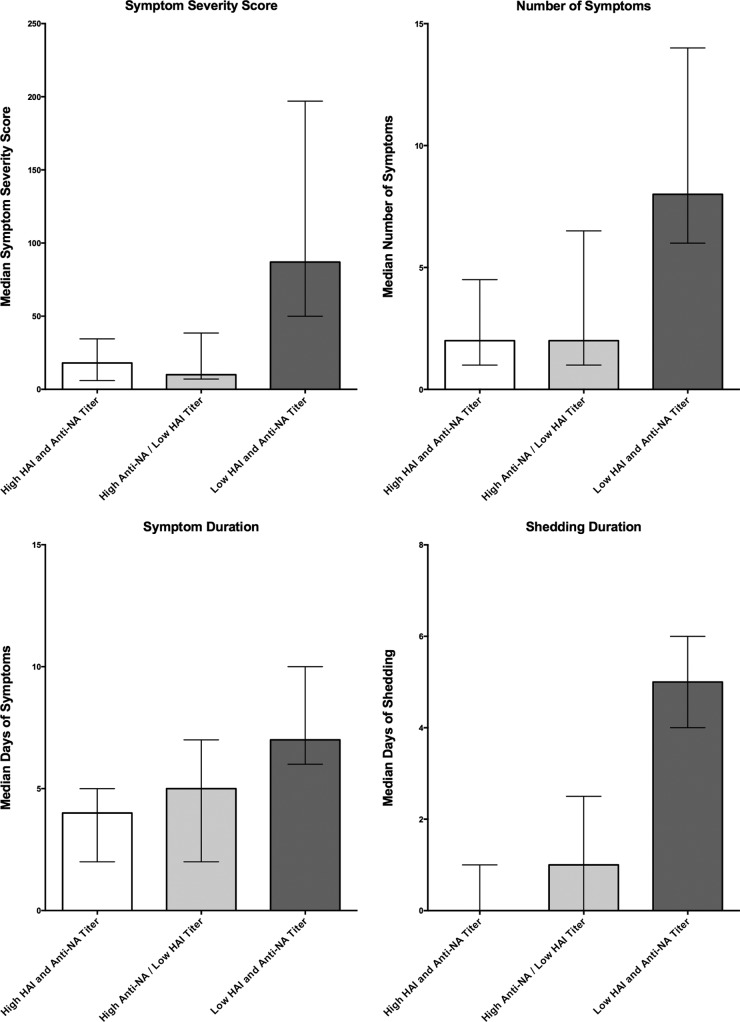
Disease severity measures in participants grouped by combinations of HAI and NAI titers. Participants were placed into three groups, those with HAI titers and NAI titers of ≥1:40 (high HAI and NAI titers; *n* = 25), those with HAI titers of <1:40 but with NAI titers of ≥1:40 (high NAI/low HAI titers; *n* = 11), and those with HAI and NAI titers of <1:40 (low HAI and NAI titers; *n* = 29). There were no individuals with low NAI and high HAI titers at baseline. For each disease metric, bars represent the median values, and error bars represent the interquartile ranges.

**FIG 6:**
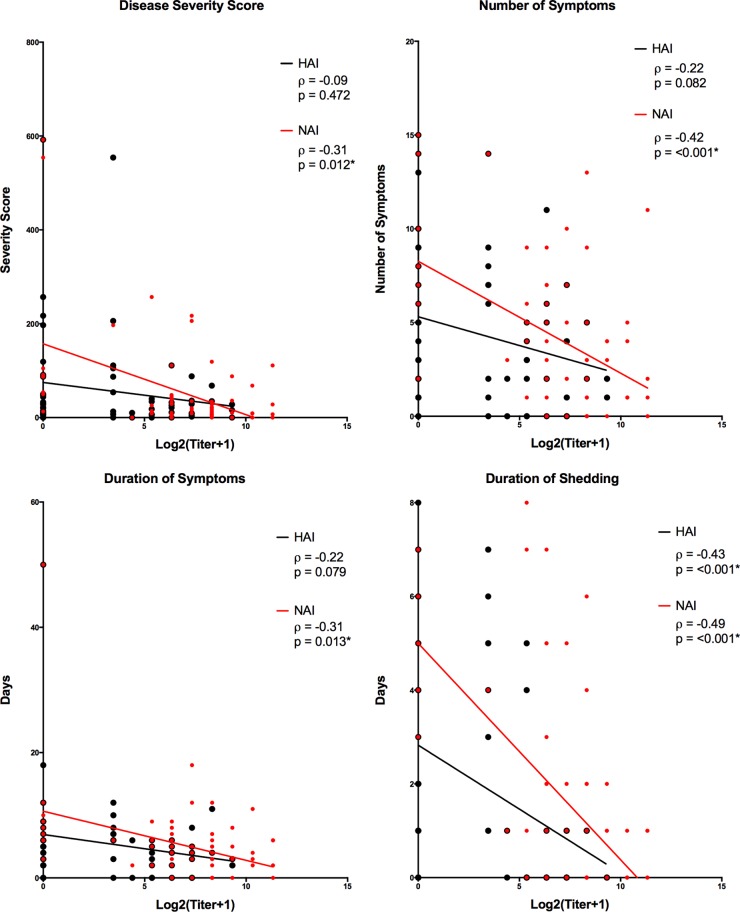
Linear correlation of baseline titer to disease severity measures. Two variable correlations were calculated using Spearman’s correlation coefficient between baseline HAI titer and all four disease severity measures (black), as well as between baseline NAI titers and all four disease severity measures (red). (Overlapping red and black points cause the red symbol to appear larger due to the presence of a black symbol in the same location.)

Overall, those participants with higher baseline levels of a combination of HAI and NAI titers had reduced disease severity over those with low titers of both HAI and NAI or of HAI alone (Fig. 5). No participants were found to have a high HAI titer with a low NAI titer. Linear regression analysis and two variable correlation analyses of baseline HAI and NAI titers demonstrated a stronger, statistically significant negative correlation between high NAI titers and reduction in all four disease severity measures compared to high baseline HAI titers. HAI titers were significantly correlated only with duration of viral shedding (Fig. 6). Multiple regression analysis was performed to evaluate the independent effects of both NAI and HAI titer on all four disease severity measures. For all four measures, increasing NAI titers demonstrated a statistically significant independent effect of decreasing severity while HAI titers showed no significant independent effect on any of the disease severity measures examined (Table 3).

**TABLE 3:** Multiple regression analysis of the contribution of baseline HAI titer and NAI titer in reduction of disease severity

Disease severity metric and variable	β	SE	*P* value^a^
Duration of shedding			
Constant	5.06105	0.55239	0
HAI titer	−0.11217	0.07961	0.164
NAI titer	−0.41608	0.08180	<0.001*
Duration of symptoms			
Constant	10.7425	1.8195	0
HAI titer	−0.1793	0.2622	0.497
NAI titer	−0.7111	0.2695	0.011*
No. of symptoms			
Constant	8.31329	1.00208	0
HAI titer	−0.9064	0.14442	0.533
NAI titer	−0.55986	0.14840	<0.001*
Symptom severity score			
Constant	156.9632	28.6784	0
HAI titer	0.4885	4.1331	0.906
NAI titer	−15.3006	4.2470	<0.001*

a Statistically significant *P* values are indicated with an asterisk.

**FIG 7:**
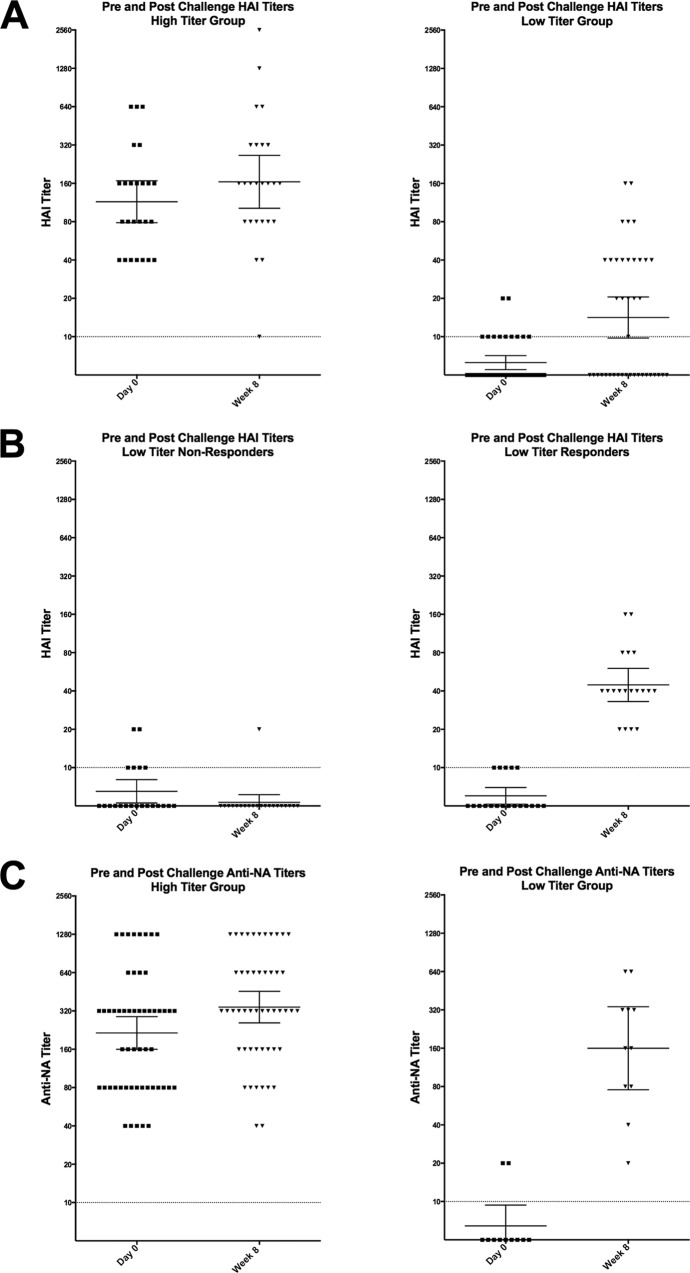
HAI and NAI titer responses postchallenge. (A) HAI titer on day 0 (prechallenge) and week 8 (postchallenge) in the participants in the low- and high-HAI-titer groups. (B) Approximately 50% of those in the low-HAI-titer group demonstrated no significant HAI response postchallenge. (C) All participants with a low NAI titer prechallenge demonstrated a significant rise in NAI titer 8 weeks postchallenge. Each symbol represents the value for an individual participant. Horizontal lines represent geometric means with 95% confidence intervals indicated by the error bars. Dotted lines represent the lower limit of detection.

Antibody responses were measured postchallenge demonstrating no significant increase in the HAI GMT in the high-HAI-titer group after challenge (week 8), but a clear increase was noted in the low-HAI-titer group (Fig. 7A). The response in the low-titer group was variable, with approximately 50% of those participants showing a very significant >4-fold rise in titer, while the other 50% of participants showed no significant increase in HAI titer (Fig. 7B). When grouped by baseline NAI titers, those participants with high titers (≥1:40) had only minimal increases in NAI GMT after challenge, but unlike HAI titer, every participant with a low NAI titer had a rise in NAI titer after challenge, regardless of clinical outcome (Fig. 7C).

## DISCUSSION

In this human influenza challenge study, participants with a prechallenge HAI titer of ≥1:40 showed significantly less incidence of MMID and some reduction in the duration of disease compared to participants with low HAI titers (<1:40) following experimental infection with a wild-type influenza A/H1N1pdm virus. These data confirm that HAI titers of ≥1:40 are indeed a correlate of protection, but while consistent with previous studies evaluating protection afforded by higher HAI titers (8–10), the results from this study help clarify that the protection predicted is not complete and that other correlates, such as NAI titer, may offer important additional or even enhanced predictive information compared to HAI titers alone. For example, in this study while high HAI titer was predictive of a reduction in the incidence of MMID, influenza symptoms without shedding were still just as likely to occur as in participants with low HAI titers. If these results are consistent with outcomes following natural influenza infection, the protection afforded by an HAI titer of ≥1:40 would not necessarily include the prevention of a clinical influenza-like illness, even if it does predict some reduction in the duration of illness and viral shedding, and thus spread of disease. This finding is important to consider for future studies and may account for some of the inconsistencies in current evaluations of influenza vaccine performance that do not take disease severity/duration into account, as many individuals receiving an efficacious vaccine might still report influenza symptoms following viral exposure/infection.

Although the incidence of influenza-like illness symptoms was not reduced in those with high HAI titers, there was a trend toward reduction of all disease severity measures, with the most significant being in duration of both viral shedding and symptoms. Overall though, many participants with high HAI baseline titers still suffered from influenza symptoms, making clear that the protection predicted by baseline HAI titers is not complete.

Interestingly, the association of high NAI titers with a more robust protective effect than HAI titers in this study cohort was striking. A reduction of disease severity in influenza infection of both animals and humans in the presence of antineuraminidase antibodies has been well described since 1969 (20–24), and as recently as 2013, it has been described as an independent predictor of reduced illness severity in natural 2009 H1N1 infection (25, 26), but this effect has never been fully evaluated in a controlled human challenge. In this study, we have not only confirmed a reduction in clinical severity but also demonstrated an independent, statistically significant effect on viral shedding, further demonstrating the strong effect antineuraminidase antibodies may have as another significant correlate of protection in influenza infection.

Not only did we find that an NAI titer of ≥1:40 was similarly predictive of reduced incidence of MMID as HAI but unlike increasing HAI titers that correlated only with reduced shedding duration, we observed that increasing NAI titers negatively correlated with all four measures of disease severity, including the duration of shedding and of symptoms, number of symptoms, and symptom severity. When evaluated together, HAI titers had no independent effect on disease severity measures including shedding duration, while NAI titers did demonstrate independent effects on all disease severity measures. Given that not all participants with high antineuraminidase titers had high HAI titers, but all those with HAI titers of ≥1:40 also had NAI titers of ≥1:40, these data suggest that the reduced disease severity observed in individuals with high baseline HAI titers is likely driven to a considerable extent by high antineuraminidase antibody titers and not an effect of antihemagglutinin antibodies alone.

Ultimately, both HAI and NAI titers together may be a better predictor of MMID and disease severity than either alone, but these data suggest that NAI titer is a stronger correlate of disease severity than is HAI titer alone. In this patient cohort, there was more variability in response after infection in HAI titers than in NAI titers during the 2 months following viral challenge. All participants with low NAI titers at baseline developed a significant increase in titer after challenge, while approximately 50% of those with low baseline HAI titers had no significant rise in titer. Those who demonstrated no rise in titer were just as likely to develop MMID and the severity of disease of these individuals was similar to those who did respond, ruling out the possibility of no infection in these cases. To the degree that a challenge model mimics natural infection, this high variability in detected antihemagglutinin antibody responses suggests that estimates of influenza infection rates based primarily on serosurveys may significantly underestimate the true infection rate and also raises concern over whether convalescent-phase HAI titers should be regarded as optimal markers of recent infection.

Influenza immunity is a complex interplay of host immunity primarily acting at the site of inoculation, the nasopharynx, and upper airway. Circulating serum antibodies to HA and NA are clear surrogate markers of immunity and may actually be a better correlate when considered together rather than separately. Although a challenge study of this type does not necessarily replicate how humans become infected with influenza naturally, it still points out the importance of careful consideration of how protection is defined and suggests a strategy by which evaluation of the correlates of protection to influenza can lead to improved evaluation of vaccine efficacy. These data further suggest that NAI titer may play a more significant role as a correlate of protection than previously thought and that the role of neuraminidase immunity should be considered when studying influenza susceptibility after vaccination and as a critical target in future influenza vaccine platforms.

Further assays on samples from this study including microneutralization, HA stalk antibody detection, immunophenotyping, and other immunologic assays will further strengthen and clarify the results observed here, and we believe it is critical to approach these questions in future laboratory studies. These data will also likely lead to future clinical studies that must be done to identify and confirm other correlates of protection beside anti-surface glycoprotein antibody titers and to develop a more refined understanding of the correlates of protection from influenza infection. Challenge studies such as this will continue to offer a well-controlled environment in which to study these factors in a unique setting where pre- and postchallenge immunity is known, the viral phenotype is controlled, timing and dose of infection are known, and serial sampling is possible.

## MATERIALS AND METHODS

### Study design.

A healthy volunteer challenge study was performed at the NIH Clinical Center after participants signed informed consent. The primary objective was to determine whether participants with a high prechallenge serum HAI titer were less likely to develop mild to moderate influenza disease (MMID) after intranasal inoculation with a wild-type influenza A/H1N1pdm virus compared to participants with a low prechallenge HAI titer. MMID was defined as viral shedding detected by clinical molecular testing, plus a minimum of one symptom of influenza after intranasal challenge, as previously described (19).

Participants with HAI titers of ≥1:40 or <1:40 prechallenge underwent a 9-day inpatient quarantine and intranasal challenge via a nasal atomizer with a 10^7^ 50% tissue culture infective dose (TCID_50_) dose of the H1N1pdm influenza challenge. Participants were assessed daily during their inpatient stay and monitored for 2 months after discharge as previously described (19). In addition, all participants performed self-assessment using the Flu-PRO (27) questionnaire twice per day for 14 days to generate a symptom severity score. The study was performed in a blind manner (all participants were unaware of their baseline HAI and NAI titers). This study (clinicaltrials.gov identifier NCT01971255) was approved by the NIAID Institutional Review Board and was conducted in accordance with the provisions of the Declaration of Helsinki and good clinical practice guidelines.

### Immunological and virological assays.

HAI titers were measured against the genetically identical challenge virus, and NAI titers were measured using an assay virus with a genetically identical NA gene compared to the challenge virus using standard methods as reported previously (9, 28). All measurements were made in triplicate. Nasal washes were analyzed for viral shedding using a one-step real-time quantitative reverse transcription-PCR (RT-PCR) for the influenza A virus matrix 1 gene (29). A standard curve with an external standard was used to calculate copy number as previously described (19).

### Statistical analysis.

The proportion with the primary outcome, MMID, was compared between the HAI titer groups in a modified intent to treat analysis (mITT) that excluded individuals who enrolled in the study and received challenge virus but were found to have a positive test for a virus other than influenza (Fig. 1). This was done to maintain the integrity of the study by eliminating any confounding infections. Analysis was also performed to evaluate the presence or absence of symptoms regardless of viral shedding. Four measures of disease severity were analyzed, including symptom severity score (Flu-PRO self-assessment), number of symptoms, symptom duration, and shedding duration. Symptom severity scores were normalized relative to baseline by subtracting prechallenge scores (maintaining a minimum score of 0). Binary outcomes were compared using the Fisher exact test, and continuous outcomes were compared using the Wilcoxon rank sum test.

In addition, we performed the same analyses after grouping participants by baseline NAI titers into groups with baseline titers of ≥1:40 and <1:40. Independent of the assigned groups, we performed two variable correlations to all four disease severity measures to both NAI and baseline HAI titers using the nonparametric Spearman’s correlation coefficient and performed multiple regression analysis using a linear model to examine independent effects of HAI and NAI titer on disease severity outcomes. All tests were two sided and at the 0.05 significance level. Statistical analyses were performed using R (version 3.0.1) (R Development Core Team, Vienna, Austria) and GraphPad Prism software (version 6.0h; GraphPad Software, La Jolla, CA).
